# Manipulating IL-2 Availability Amid Presentation of Donor MHC Antigens Suppresses Murine Alloimmune Responses by Inducing Regulatory T Cells

**DOI:** 10.1371/journal.pone.0008756

**Published:** 2010-01-18

**Authors:** Shuzi Zhang, Hehua Dai, Ni Wan, Yolonda Moore, Zhenhua Dai

**Affiliations:** Center for Biomedical Research, University of Texas Health Science Center, Tyler, Texas, United States of America; New York University, United States of America

## Abstract

**Background:**

Major histocompatibility complex (MHC) antigens are important for alloimmune responses as well as immune tolerance. Previous studies have shown that presentation of donor MHC antigens by donor-specific transfusion prior to or upon transplantation promotes transplant tolerance induced by other agents. However, it is unclear whether presentation of donor MHC antigens by DNA vaccination induces long-term allograft survival.

**Methodology/Principal Findings:**

We investigated whether presentation of MHC class-II and/or class-I donor antigens by DNA vaccination suppresses alloimmune responses and promotes long-term allograft acceptance. We initially found that presentation of both MHC donor antigens by DNA vaccination itself prior to transplantation fails to significantly prolong islet allograft survival in otherwise untreated mice. However, islet allograft survival was significantly prolonged when MHC class-II DNA vaccination was accompanied with IL-2 administration (MHCII + IL-2) while MHC class-I DNA vaccination was followed by IL-2 and subsequent neutralizing anti-IL-2 treatments (MHCI + IL-2/anti-IL-2). Especially, this protocol promoted long-term allograft survival in the majority of recipients (57%) when combined with low doses of rapamycin post-transplantation. Importantly, MHCII + IL-2 induced FoxP3+ Treg cells in both spleens and grafts and suppressed graft-infiltrating CD4+ cell proliferation, whereas MHCI + IL-2/anti-IL-2 mainly inhibited graft-infiltrating CD8+ cell proliferation and donor-specific CTL activity. The combined protocol plus rapamycin treatment further reduced both CD4+ and CD8+ T cell proliferation as well as donor-specific CTL activity but spared FoxP3+ Treg cells. Depleting CD25+ Treg cells or adoptive transfer of pre-sensitized CD8+ T cells abolished this long-term allograft survival.

**Conclusions/Significance:**

Manipulating IL-2 availability during presentation of MHC class-II and class-I donor antigens by DNA vaccination pre-transplantation induces Treg cells, suppresses alloimmune responses and promotes long-term allograft survival.

## Introduction

A transplanted organ or islet is always rejected without immunosuppressive treatments. However, treatments with immunosuppressive drugs usually cause severe side-effects including viral infection and tumors. An approach to inducing long-term allograft survival or tolerance without long-term immunosuppression after transplantation is highly desired in the field. Current main strategies to promote long-term graft survival or tolerance include donor-specific transfusion, T cell costimulatory blockade and induction of Treg cells. In particular, the presentation of donor MHC antigens to recipients prior to or upon transplantation, though does not generally prolong allograft survival by itself, promotes long-term allograft survival or tolerance induced by additional treatments including CD40/CD154 or CD28/B7 costimulatory blockade. This presentation of donor MHC antigens has been largely carried out by donor-specific transfusion (DST) [1]–[4], MHC allopeptides recognized in the thymus [5], and transgenic expression of donor MHC antigens in bone marrow cells, resulting in mixed chimerism [6], [7]. However, these measures require a live donor for the source of donor-derived blood cells and complex models such as bone-marrow chimerism, and are not practical for clinical applications. Moreover, T cell costimulatory blockade fails to consistently induce transplant tolerance in many animal models.

IL-2, primarily produced by activated T cells, is a major effector cytokine that mediates immunity and inflammatory responses including allograft rejection. However, it also promotes activation-induced T cell death [8], [9] and is essential for CD4+CD25+ Treg cell development and homeostasis [10]–[13]. Therefore, IL-2 is indispensable for the induction of long-term allograft survival or tolerance [14]–[18].

In this study, we sought to study whether presentation of both MHC class II and class I donor antigens by DNA vaccination promotes long-term allograft acceptance, since cadaverous donor MHC genes can be still constructed. We initially found that the presentation of MHC class II and/or class I donor antigens by DNA vaccination alone fails to significantly prolong islet allograft survival in immune competent wild-type mice. We then sought to take advantage of the redundant features of IL-2 and promote long-term allograft survival by inducing Treg cells. In these experiments, we investigated whether manipulating IL-2 availability during presentation of MHC class II and/or class I donor antigens by DNA vaccination prior to transplantation suppresses alloimmune responses. We found that administration of IL-2 following MHC class-II DNA vaccination prior to transplantation induces FoxP3+ Treg cells in both spleens and allografts and suppresses graft-infiltrating CD4+ cell proliferation, and that MHC class-I DNA vaccination, followed by IL-2 administration and subsequent neutralizing anti-IL-2 treatment pre-transplantation, inhibits graft-infiltrating CD8+ T cell proliferation and donor-specific CTL activity. The combined treatment protocol with both MHC class-II and class-I DNA vaccination and manipulating IL-2 availability prior to transplantation significantly prolonged islet allograft survival in the absence of any subsequent immunosuppressive treatment post-transplantation. More importantly, this protocol induced long-term islet allograft survival in the majority of recipient mice when further combined with an additional treatment with low doses of rapamycin post-transplantation.

## Results

### Presentation of Both MHC Class-II and Class-I Donor Antigens by DNA Vaccination Prior to Transplantation Promotes the Induction of Long-Term Islet Allograft Acceptance

**Figure 1:**
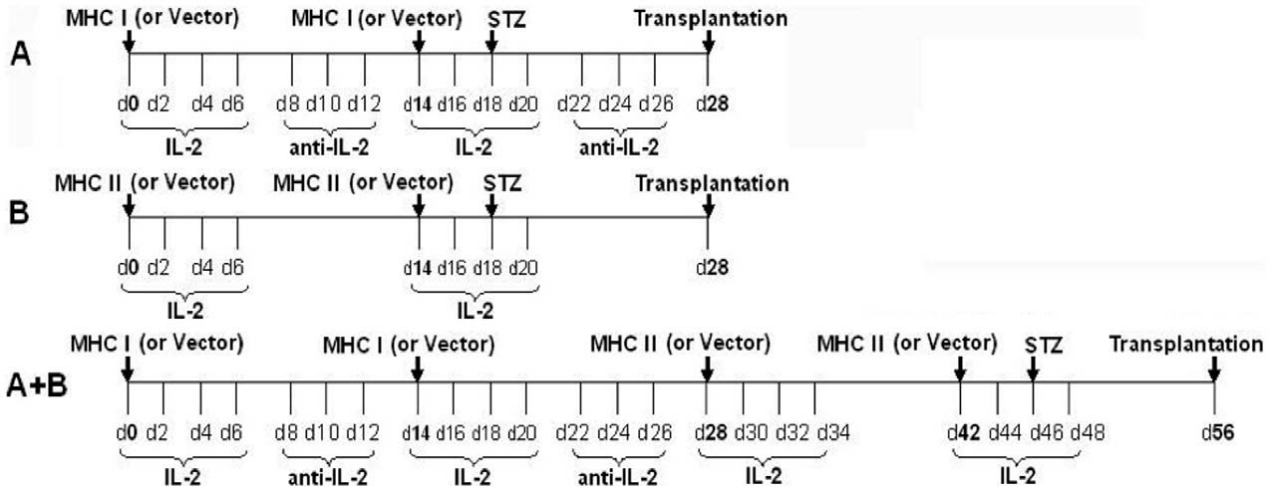
MHC immunization procedure. Mice were immunized with donor MHC I and/or MHC II DNA vaccines and treated with recombinant IL-2 and/or anti-IL-2 Ab as well as streptozotocin (STZ) before islet transplantation.

**Figure 2:**
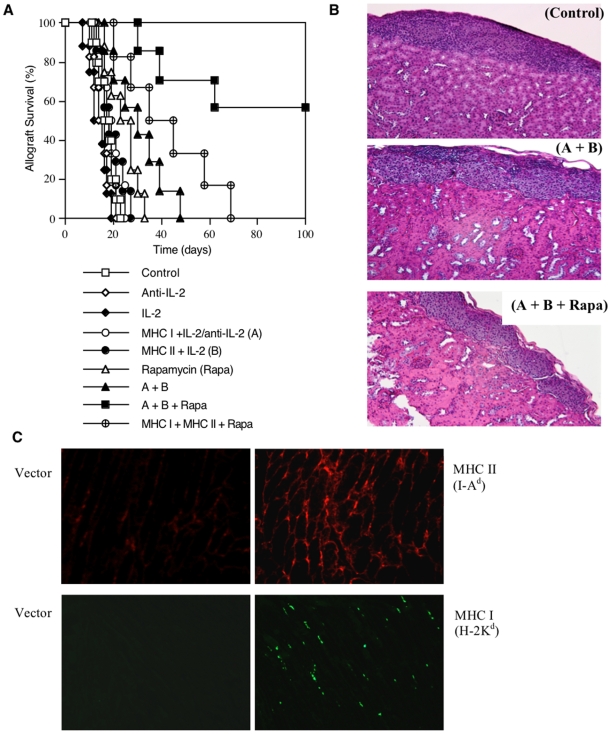
Donor MHC DNA vaccination and administration of IL-2 regimen prior to transplantation promote long-term islet allograft survival. Prior to transplantation, B6 mice were untreated (□, n = 10) or treated with neutralizing anti-IL-2 (◊, n = 6) alone, recombinant IL-2 alone (υ, n = 8), donor MHC class-I DNA vaccination plus both IL-2 and subsequent anti-IL-2 (MHC I + IL-2/anti-IL-2: A) (○, n = 6), donor MHC class-II DNA vaccination plus IL-2 (MHC II + IL-2: B) (λ, n = 7), or both MHC I and MHC II vaccination plus IL-2 regimen (A + B) (σ, n = 7). In some groups, rapamycin control mice (Rapa) (▵, n = 8) or mice treated with both MHC vaccinations and IL-2 regimen (A + B + Rapa) (ν, n = 7) or both vaccinations without IL-2 (MHC I + MHC II + Rapa) (⊕, n = 6) received low doses of rapamycin post-transplantation. (**A**). Islet allograft rejection was observed. (**B**). H&E staining on kidney sections at the time of rejection or 100 days after transplantation. (**C**). Immunofluorescence staining on muscular frozen sections for the expression of donor MHC I (H-2K^d^) or MHC II (I-A^d^) three days after donor MHC DNA vaccination in a recipient. One representative of three independent experiments is shown.

To study whether presentation of both MHC class-II and class-I donor antigens by DNA vaccination promotes long-term allograft survival, B6 mice were immunized with MHC class-II and/or class-I donor antigens by DNA vaccines and treated with recombinant IL-2 or neutralizing anti-IL-2 Ab, as described in the Methods section and **Figure 1**, before they received islet allografts from Balb/C donors. As shown in **Figure 2A**, neither MHC I + IL-2/anti-IL-2 nor MHC II + IL-2 treatment significantly prolonged islet allograft survival compared to control group (median survival time, MST = 18 vs. 16 days or 19 vs. 16 days, both P>0.05). Also as controls, neither anti-IL-2 alone nor IL-2 alone prior to transplantation altered islet allograft survival (MST = 15 vs. 16 days or 14 vs. 16 days, both P>0.05). Treatments with isotype Ab for anti-IL-2 or with MHC I and/or MHC II immunization but without IL-2 did not affect allograft survival (data not shown in the figure). However, the protocol that combined MHC I + IL-2/anti-IL-2 and subsequent MHC II + IL-2 treatments (A + B) significantly prolonged islet allograft survival compared to control group (MST = 31 vs. 16 days, P<0.05). More importantly, this combined protocol induced long-term islet allograft survival in the majority of recipients (57%) when rapamycin, at low doses, was also administered after transplantation (A+ B+ Rapa). On the other hand, treatments with the same doses of rapamycin failed to induce long-term islet allograft survival in the presence of either MHC I + IL-2/anti-IL-2 (A) or MHC II + IL-2 B (data not shown in the figure). Although rapamycin plus both MHC I and MHC II vaccination without the IL-2 regimen (MHC I + MHC II + Rapa) significantly prolonged allograft survival compared to control group (MST = 40 vs. 16 days, P<0.05), they did not induce long-term allograft survival and were much less effective than the same treatments plus IL-2 regimen (A+ B+ Rapa) (MST = 40 vs. 112 days, P<0.05), suggesting that IL-2 regimen in this treatment protocol is essential for long-term allograft survival. H&E staining on sections of the kidney confirmed cellular infiltration in and around islet grafts in the control group (Control) and the group treated with MHC I + IL-2/anti-IL-2 plus MHC II + IL-2 (A + B) but not the group treated with A + B + Rapa (**Figure 2B**) that achieved long-term allograft survival. Moreover, tissue immunofluorescence staining also confirmed the expression of donor MHC class-II (I-A^d^) and MHC class-I (H-2K^d^) molecules three days after DNA vaccination (**Figure 2C**).

On the other hand, the combined treatments with both donor MHC DNA vaccination and IL-2 regimen as well as low doses of rapamycin (A + B + Rapa) also induced long-term islet engraftment in 63% of C3H/HeJ recipients (n = 8, data not shown). Taken together, our findings suggest that manipulating IL-2 availability during presentation of both MHC class-II and class-I donor antigens by DNA vaccination prior to transplantation suppresses allograft rejection and induces long-term allograft survival in the presence of mild immunosuppression with low doses of rapamycin.

### Donor MHC DNA Vaccination and Administration of IL-2 Prior to Transplantation Induce FoxP3+ Treg Cells

**Figure 3:**
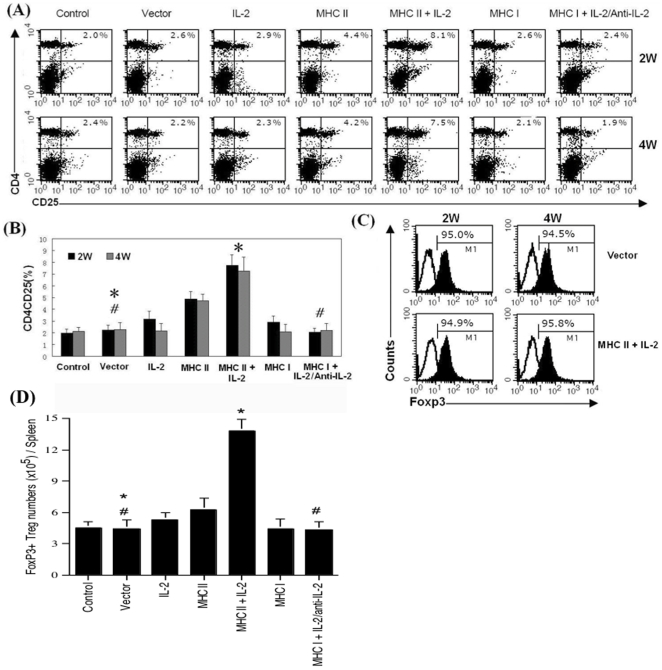
Donor MHC II DNA vaccination and administration of IL-2 prior to transplantation induce FoxP3+ Treg cells. B6 mice were treated with recombinant IL-2 alone, anti-IL-2 alone, immunization with vector, MHC I or MHC II alone, or both IL-2 regimen and donor MHC DNA vaccination in recipients as indicated. Two and four weeks after secondary immunization, spleen cells were stained for CD4, CD8 and FoxP3 expression and analyzed by FACS. (**A**). Density plot is shown for the percentage of CD4+CD25+ Treg cells from one representative experiment. (**B**). Data are presented as mean ± SEM and represent an average of three independent experiments (*P<0.05 and ^#^P>0.05). (**C**). Histograms shown are percentages of FoxP3+ cells after gating on CD4+CD25+ cells from control group. One representative of three experiments is shown. (**D**). The absolute numbers of FoxP3+ Treg cells per spleen two weeks after final immunization were also calculated (*P<0.05 and ^#^P>0.05).

IL-2 is essential for CD4+CD25+ Treg cell development and homeostasis [10]–[12]. To study whether IL-2 manipulation and presentation of both MHC class-II and class-I donor antigens by DNA vaccination prior to transplantation suppress allograft rejection by inducing Tregs, we first measured CD4+CD25+FoxP3+ Treg cells from spleens of B6 mice, which were immunized and/or treated with IL-2/antiIL-2 regimen without transplantation, two and four weeks after second immunization as described in the section of Methods. Based on one representative FACS data, as shown in **Figure 3A**, immunization with MHC II-expressing DNA alone increased the percentages of Treg cells (4.4 vs. 2.5% at 2 weeks and 4.2 vs. 2.2% at 4 weeks) that were further increased when IL-2 was also administered (8.1 vs. 2.5% at 2 weeks and 7.5 vs. 2.2% at 4 weeks) while immunization with MHC I-expressing DNA alone or in combination with IL-2/anti-IL-2 did not increase Treg cells. Based on three separate experiments in which data are presented as mean ± SEM as shown in **Figure 3B**, we confirmed that MHC II DNA immunization alone significantly increased Treg cells compared to vector control (2 weeks: 4.9±0.6% vs. 2.2±0.4%, and 4 weeks: 4.7±0.5% vs. 2.3±0.6%, both P<0.05) while addition of IL-2 further increased Treg cells compared to MHC II DNA immunization alone (2 weeks: 7.8±0.8% vs. 4.9±0.6%, and 4 weeks: 7.2±1.1 vs. 4.7±0.5%, both P<0.05). IL-2 alone slightly increased Treg cells at the time point of 2 weeks (3.1±0.6% vs. 2.0±0.4%, P = 0.054) but not 4 weeks (2.2±0.6% vs. 2.1±0.3%, P>0.05). Moreover, MHC I immunization alone or in combination with IL-2 (data not shown) or with IL-2 plus subsequent anti-IL-2 did not significantly alter Treg numbers (Figure 3B). Around 95% of CD4+CD25+ cells were confirmed to be FoxP3+ Treg cells (**Figure 3C**). Finally, as shown in **Figure 3D**, MHC II + IL-2 also significantly increased the absolute number of FoxP3+ cells in the spleen two weeks after last immunization (13.8±1.1 vs. 4.4±0.9, ×10^5^/spleen, P<0.05) while MHC I + IL-2/anti-IL-2 did not (4.3±0.8 vs. 4.4±0.9, ×10^5^/spleen, P>0.05).

**Figure 4:**
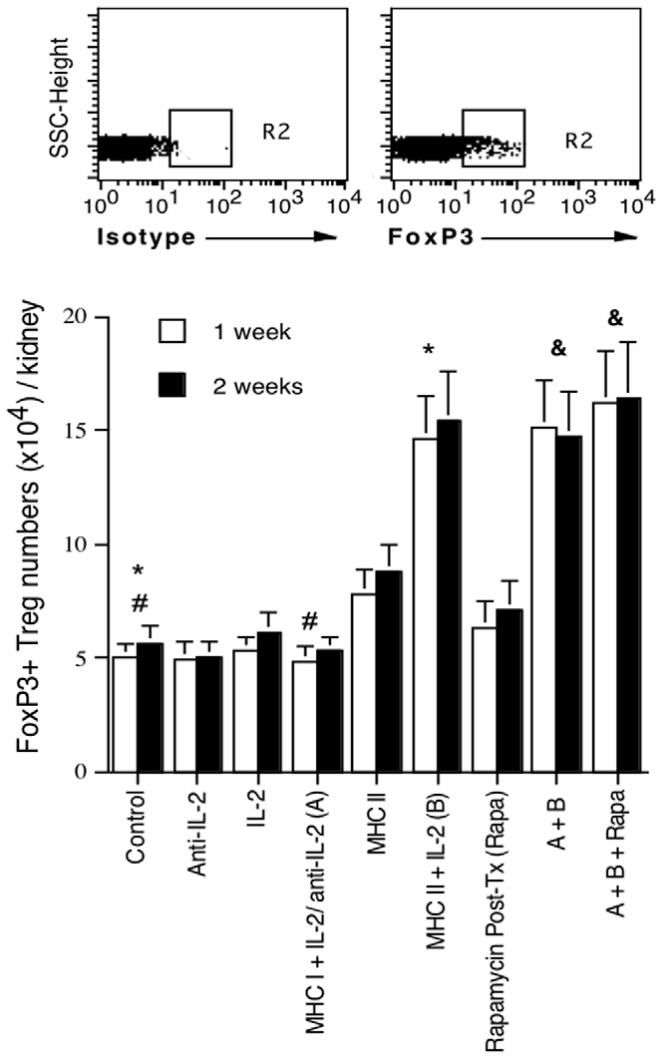
The absolute number of FoxP3-positive Treg cells in allografts. Prior to transplantation, B6 mice were immunized with donor MHC DNA vaccines and/or treated with IL-2 regimen as described in Figure 1. One and two weeks after receiving Balb/C islets under the kidney capsule, recipient mice were sacrificed and graft-infiltrating cells were isolated and stained for FoxP3 to quantify FoxP3+ Treg cells by FACS. FoxP3+ cell numbers were calculated by the formula that total cell numbers per kidney time the percentage of FoxP3+ cells (%). Results are shown as mean ± SEM from four independent experiments (*P<0.05, ^#^P>0.05, and ^&^P>0.05).

To further quantify Treg cells in grafts after transplantation, mice that were immunized and/or treated with IL-2/anti-IL-2 were sacrificed one and two weeks after islet transplantation, and graft-infiltrating cells were isolated and analyzed to determine the absolute numbers of FoxP3+ Treg cells per kidney. As shown in **Figure 4**, treatments with IL-2, anti-IL-2, or together with MHC I DNA vaccination prior to transplantation did not alter the numbers of Treg cells in grafts one week after islet transplantation. Neither did the vector control (data not shown in the figure). However, MHC II DNA vaccination significantly increased Treg cell numbers compared to control group (7.8±1.1 vs. 5.0±0.5, P<0.05) while Treg numbers were further increased in MHC II + IL-2 group compared to MHC II alone (14.6±1.9 vs. 7.8±1.1, P<0.05) one week after transplantation. Administration of rapamycin alone post-transplantation slightly but not significantly increased the numbers of Tregs in the grafts compared to the control (6.3±1.2 vs. 5.0±0.5, P>0.05). Rapamycin also did not significantly alter Treg numbers when combined with MHC II + MHC I plus IL-2 regimen (A+ B+ Rapa vs. A+ B: 16.2±2.3 vs. 15.1±2.1, P>0.05). The similar results were obtained two weeks after transplantation (**Figure 4**). Taken together, MHC II DNA vaccination alone or together with administration of IL-2 prior to transplantation dramatically increased the number of Treg cells in both spleens and grafts of recipients while addition of rapamycin after transplantation did not significantly alter the numbers of Treg cells in grafts. These findings suggest that donor MHC class-II, but not MHC class-I, DNA vaccination induces Treg cells and is more efficient when additional IL-2 is available.

### Donor MHC DNA Vaccination and Manipulating IL-2 Availability prior to Transplantation Suppress Graft-Infiltrating T Cell Proliferation

**Figure 5:**
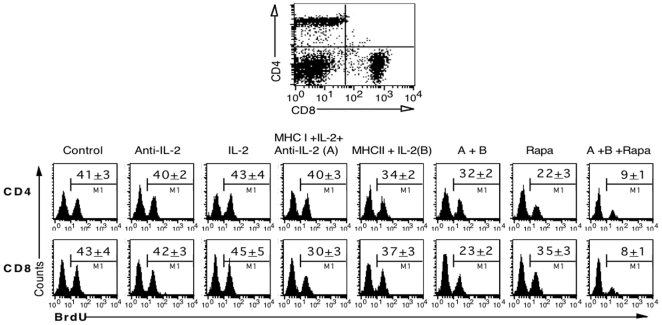
In vivo proliferation of graft-infiltrating T cells. Prior to transplantation, B6 mice were immunized with donor MHC DNA vaccines and/or treated with IL-2 regimen as described in Figure 1 legend. One week after receiving Balb/C islets under the kidney capsule, graft-infiltrating cells from B6 recipients were isolated to determine CD4+ and CD8+ T cell proliferation by BrdU uptakes. Histograms are shown after gating on CD4+ or CD8+ population. One representative histogram per group from three separate experiments is shown, and data are presented as mean ± SEM from three independent experiments. MHC II + IL-2 mainly suppressed CD4+ T cell proliferation while MHC I + IL-2/anti-IL-2 inhibited CD8+ T cell proliferation. The treatment protocol combining both suppressed both CD4+ and CD8+ cell proliferation, and further prevented T cell proliferation when rapamycin, at low doses, was additionally administered.

To study how donor MHC DNA vaccination and manipulating IL-2 suppress alloimmune responses, mice that were immunized and/or treated with IL-2 regimen prior to transplantation were sacrificed one week after islet transplantation, and graft-infiltrating cells from the kidney harboring islet allografts were isolated to measure T cell proliferation by BrdU uptakes. As shown in **Figure 5**, treatment with IL-2 or anti-IL-2 alone prior to transplantation did not alter the percentages of BrdU-positive cells in both CD4+ and CD8+ components one week after transplantation (BrdU+ in anti-IL-2 group: CD4: 40±2% vs. 41±3%, CD8: 42±3% vs. 43±4%; and BrdU+ in IL-2 group: CD4: 43±4% vs. 41±3%, CD8: 45±5% vs. 43±4%, all P>0.05). Neither did the vector control, MHC II alone, MHC I alone or MHC I + anti-IL-2 (data not shown in the figure). However, MHC I + IL-2/anti-IL-2 (**A**) pre-transplantation significantly suppressed CD8+, but not CD4+, T cell proliferation (CD4: 40±3% vs. 41±3%, P>0.05 and CD8: 30±3% vs. 43±4%, P<0.05), whereas MHC II + IL-2 (**B**) mainly inhibited CD4+ cell proliferation and only slightly inhibited CD8+ cell proliferation (CD4: 34±2% vs. 41±3%, P<0.05 and CD8: 37±3% vs. 43±4%, P>0.05). Moreover, treatments with both MHC II and MHC I plus IL-2 regimen (**A+B**) suppressed both CD4+ and CD8+ cell proliferation (CD4: 32±2% vs. 41±3%, P<0.05 and CD8: 23±2% vs. 43±4%, P<0.05). On the other hand, low dose of rapamycin alone post-transplantation without immunization pre-transplantation significantly suppressed CD4+ cell proliferation but only slightly inhibited CD8+ cell proliferation (CD4: 22±3% vs. 41±3%, P<0.05 and CD8: 35±3% vs. 43±4%, P = 0.056). Importantly, the combined treatments with MHC I/II vaccination, IL-2 regimen and rapamycin (**A+ B+ Rapa**) largely prevented both CD4+ and CD8+ cell proliferation (CD4: 9±1% vs. 41±3%, P<0.05 and CD8: 8±1% vs. 43±4%, P<0.05, **Figure 5**). Taken together, MHC I + IL-2/anti-IL-2 pre-transplantation mainly suppressed CD8+ T cell proliferation in the grafts, whereas MHC II + IL-2 pre-transplantation mainly inhibited CD4+ T cell proliferation. The measures combining both suppressed both CD4+ and CD8+ T cell turnover and largely prevented T cell proliferation when rapamycin, at low doses, was also administered post-transplantation.

### Donor MHC Class-I DNA Vaccination and Treatment with IL-2 Regimen prior to Transplantation Reduce Donor-Specific Cytotoxic T Lymphocyte (CTL) Activity

**Figure 6:**
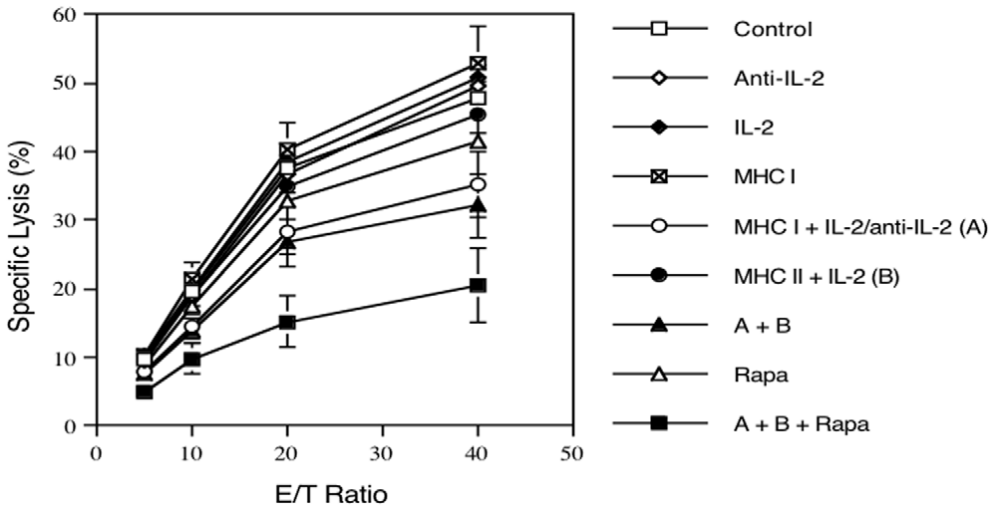
Donor-specific CTL activity of graft-infiltrating cells. One week after transplantation with Balb/C islets, graft-infiltrating cell were isolated from B6 recipient mice that were immunized with donor MHC DNA vaccines and/or treated with IL-2 regimen prior to transplantation, and analyzed for donor-specific CTL activity. MHC I+IL-2/anti-IL-2, but not MHC II+IL-2, treatments significantly suppressed CTL activity while the treatments with both plus rapamycin further reduced donor-specific CTL activity.

Since donor MHC I DNA vaccination plus IL-2 regimen prior to transplantation suppressed the proliferation of graft-infiltrating CD8+ T cells (Figure 4), we asked whether they also affect donor-specific CTL activity ex vivo of graft-infiltrating cells in B6 recipient mice one week after islet transplantation. As shown in **Figure 6**, treatment with anti-IL-2 alone, IL-2 alone or MHC II + IL-2 (**B**) prior to transplantation did not alter the CTL activity of graft-infiltrating cells. MHC I vaccination alone slightly, but not significantly, increased CTL activity (E/T of 5: 10.3±0.7 vs. 9.8±0.5; 10: 21.4±2.3 vs. 19.5±2.1; 20: 40.2±3.9 vs. 37.7±3.6; and 40: 52.8±5.6 vs. 47.7±4.9, P>0.05). However, MHC I + IL-2/anti-IL-2 (**A**) significantly suppressed CTL activity (E/T of 5: 8.0±0.4 vs. 9.8±0.5; 10: 14.5±1.6 vs. 19.5±2.1; 20: 28.4±3.2 vs. 37.7±3.6; and 40: 35.2±4.8 vs. 47.7±4.9, P<0.05). The treatment combining both MHC I and MHC II as well as IL-2 regimen (**A+B**) further inhibited CTL activity (P<0.05, Figure 5) and exhibited a maximal reduction in CTL activity when rapamycin, at low doses, was additionally used (**A+B+Rapa**) (E/T of 5: 4.9±0.4 vs. 9.8±0.5; 10: 9.7±2.2 vs. 19.5±2.1; 20: 15.3±3.7 vs. 37.7±3.6; and 40: 20.5±5.4 vs. 47.7±4.9, P<0.05), whereas rapamycin alone, at the low doses, was not sufficient to significantly suppress donor-specific CTL activity (Figure 5). As controls, neither MHC II plus IL-2/anti-IL-2 nor MHC I plus anti-IL-2 without IL-2 significantly altered CTL activity (data not shown). Taken together, MHC I, but not MHC II, DNA vaccination plus IL-2 regimen prior to transplantation significantly reduced donor-specific CTL activity of graft-infiltrating cells and worked more efficiently when combined with low doses of rapamycin.

### Depleting CD25+ Treg Cells or Adoptive Transfer of Pre-Sensitized CD8+ T Cells Reverses Long-Term Allograft Survival Induced by Donor MHC DNA Vaccination and IL-2 Regimen

**Figure 7:**
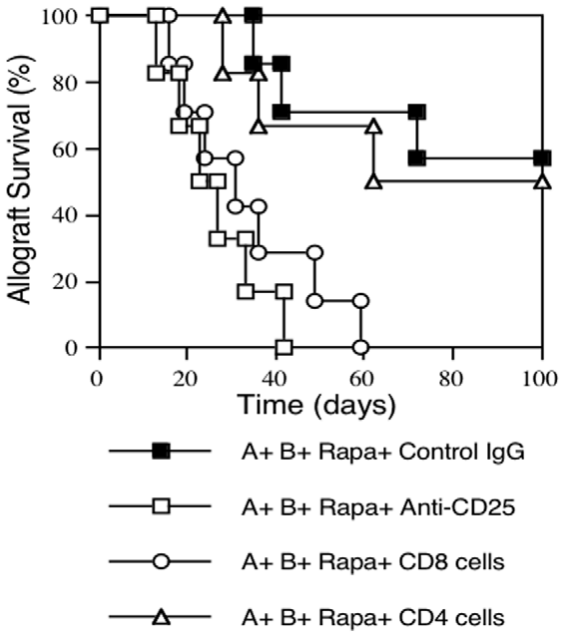
Depleting CD25+ Treg cells or adoptive transfer of pre-sensitized CD8+ T cells abrogates long-term islet allograft survival. B6 mice were treated with MHC I + IL-2/anti-IL-2 and subsequent MHC II + IL-2 prior to transplantation, transplanted with Balb/C islets, and treated with low doses of rapamycin post-transplantation (A + B + Rapa). They were also treated with either depleting anti-CD25 Ab (A + B + Rapa + Anti-CD25) (□, n = 6) or control IgG (A + B + Rapa + Control IgG) (ν, n = 7), or received upon transplantation syngeneic CD8+CD25− (A + B + Rapa + CD8) (○, n = 7) or CD4+CD25− T cells (A + B + Rapa + CD4) (▵, n = 6) that were isolated from B6 mice pre-sensitized *i.p*. with irradiated Balb/C splenocytes two weeks earlier. Depleting CD25+ Treg cells or adoptive transfer of pre-sensitized CD8+, but not CD4+, T cells reversed long-term islet allograft survival induced by treatments with donor MHC DNA vaccination and IL-2 regimen prior to transplantation plus rapamycin treatment post-transplantation.

Since donor MHC DNA vaccination and IL-2 regimen prior to transplantation induced Treg cells and promoted long-term allograft survival, we then asked whether this long-term allograft survival is dependent on Treg cells. The immunized B6 mice that also received IL-2 regimen prior to transplantation, as described in Methods section, were transplanted with Balb/C islets and treated with low dose of rapamycin as well as depleting anti-CD25 Ab or isotype control. As shown in **Figure 7**, we found that depleting CD25+ Treg cells abrogated the long-term islet allograft survival induced by MHC DNA vaccination plus IL-2 regimen pre-transplantation and the rapamycin treatment post-transplantation (MST = 25 days). Moreover, this long-term allograft survival could not be achieved at all in IL-2-deficient mice that lack CD25+ T cells (data not shown). On the other hand, the long-term allograft survival induced by this protocol was also reversed by the adoptive transfer of CD8+CD25− T cells that were isolated from mice that were sensitized i.p. with Balb/C splenocytes two weeks prior to the cell transfer (MST = 31 days). However, transfer of pre-sensitized CD4+CD25− counterparts did not significantly shorten allograft survival (MST = 92 vs. 102 days, P>0.05). These findings suggest that long-term allograft survival, induced by rapamycin post-transplantation and donor MHC DNA vaccination plus IL-2 regimen pre-transplantation, is dependent on CD25+ Treg cells and that adoptive transfer of allo-sensitized CD8+, but not CD4+, T cells prevents this long-term allograft survival.

## Discussion

Using islet allograft rejection as readout, we investigated the impact of IL-2 availability amid donor MHC DNA vaccination in recipients pre-transplantation on alloimmune responses. We found that manipulating IL-2 availability during presentation of donor MHC class-II and class-I antigens by DNA vaccination prior to transplantation induces Treg cells, suppresses alloimmune responses and promotes the induction of long-term allograft survival. In this study we utilized two approaches that may have significant advantages over previous protocols for inducing long-term allograft survival. Firstly, donor MHC DNA vaccination, as conducted in this study, has some advantages over donor-specific blood or splenocyte transfusion (DST) that is inconvenient and not available in the case of cadaveric organ transplantation. Secondly, administration of IL-2, together with donor MHC DNA vaccination prior to transplantation, induced Treg cells and helped prolong allograft survival in this study while previous studies demonstrated that treatments with IL-2, an effector cytokine, upon or after transplantation generally enhanced effector T cell function and accelerated allograft rejection [19]. Therefore, the timing for IL-2 treatments is critical for enhancing allograft survival. Thus, our findings may have clinical implications for inducing long-term allograft survival in transplanted patients.

It appears that presentation of donor MHC antigens alone in recipients prior to transplantation is not sufficient to prolong allograft survival, as our data have shown that presentation of both MHC class-I and class-II donor antigens by DNA vaccination alone without additional IL-2 manipulation prior to transplantation fails to significantly prolong allograft survival. This is consistent with many of the recent studies demonstrating that presentation of MHC donor antigens by DST alone was incapable of prolonging allograft survival in experimental animals, although it did induce long-term allograft survival or tolerance when combined with additional treatments [1], [2], [20]–[22]. However, presentation of MHC II donor antigens or allopeptides alone in the unique environment, including the bone marrow or thymus [5], [6], promoted long-term allograft acceptance via bone marrow chimerism or thymic selection. Moreover, a previous pioneering study by Peche et al has also shown that donor MHC class-II DNA vaccination alone can delay acute allograft rejection in experimental rats [23]. The discrepancy between those results and ours may be attributed to different animal models and locations of donor MHC antigen presentation as well as the loci of MHC genes. Interestingly, previous studies have demonstrated that a MHC class II-derived soluble peptide, HLA-DQA1, inhibits alloimmune responses by binding to the host MHC class II molecule outside of the MHC-binding groove [24]. This peptide altered T cell-APC interaction, promoted host APC apoptosis [25], [26] and induced Treg cells [27]. These interesting studies, though with similar transplantation outcomes, were conducted using totally different approaches from ours. We utilized MHC DNA constructs that expressed intact donor MHC molecules, which generally invoke alloimmune responses in recipients in the absence of other immune-based manipulations, while MHC class II-derived soluble peptide directly interfered with alloimmune responses by altering T cell-APC interaction and APC apoptosis [25], [26]. On the other hand, an important study by Chiang et al have identified a novel peptide as an immunosuppressive vaccine for the prolongation of allograft survival [28], further indicating that vaccination may hold promise for inducing long-term allograft survival or tolerance.

In this current study we found that donor MHC II vaccination plus administration of IL-2 prior to transplantation induced Treg cells but did not significantly prolong allograft survival, suggesting that induction of Treg cells alone is insufficient to prolong allograft survival in WT mice. On the other hand, treatments with donor MHC I vaccine plus recombinant IL-2 and subsequent anti-IL-2 Ab pre-transplantation inhibited graft-infiltrating CD8+ cell proliferation and donor-specific CTL activity, but failed to significantly prolong allograft survival, indicating that reduction in allospecific CD8+ cell proliferation and their function is also not sufficient to prolong allograft survival. The treatments combining both MHC II and MHC I donor antigen vaccination as well as IL-2 manipulation (A + B) significantly prolonged allograft survival, because they were capable of both inducing Treg cells and suppressing CD8+ T cell function. Moreover, the combined protocol induced long-term allograft survival in most of the recipients when rapamycin, at low doses, was also administered (A + B + Rapa). Accordingly, addition of low doses of rapamycin to the combined protocol further enhanced the suppression of graft-infiltrating CD4+ and CD8+ T cell proliferation and donor-specific CTL activity but preserved FoxP3+ Treg cells. Our protocols using IL-2 regimen differ from others in that recombinant IL-2 and anti-IL-2 Ab (clone JES6-1A12) were administered separately in our experiments. The rationale for using IL-2 and subsequent anti-IL-2 treatments is that IL-2 generally promotes activation-induced T cell death (AICD) [8], [9], the active cell death, and that subsequent anti-IL-2 treatments neutralize IL-2, a growth factor, and promote passive T cell death via “IL-2 withdrawal”. It is anticipated that both active and passive T cell death would help reduce donor-reactive T cell numbers before transplantation. Indeed, we found that donor MHC I vaccination plus IL-2 and subsequent anti-IL-2 Ab treatments pre-transplantation inhibit graft-infiltrating CD8+ cell proliferation and donor-specific CTL activity. Studies by Webster et al utilized a unique IL-2/anti-IL-2 complex that was designed to extend the half-life of the exogenous IL-2 [29]. Their IL-2 component was able to induce Treg cells but did not directly suppress CD8+ T cell response [17].

CD4^+^CD25^+^ regulatory T (Treg) cells play a key role in the maintenance of immune tolerance to both self- and foreign-antigens by suppressing aggressive T cell responses. Treg cells represent a small fraction (5–10%) of CD4+ T cells and constitutively express the α chain of the IL-2 receptor (CD25) [30] and the CTLA-4 [31], [32]. The induction of endogenous Treg cells or adoptive transfer of exogenous Treg cells prevented autoimmune diseases and allograft rejection in many animal models [33]–[50]. IL-2 promotes activation-induced T cell death (AICD) [8], [9] and is essential for CD4+CD25+ Treg cell development and homeostasis [10]–[13] as well as the induction of long-term allograft survival or tolerance [14]–[18]. Hence, blocking IL-2 after transplantation could hinder tolerance, although it did suppress effector T cells and prolonged allograft survival [51]. Here we found that donor MHC II DNA vaccination plus recombinant IL-2 induced Treg cells. These Treg cells were essential for long-term allograft survival induced by the combined protocol plus low doses of rapamycin, because depleting CD25+ Treg cells abrogated this long-term allograft survival. On the other hand, donor MHC I DNA vaccination plus recombinant IL-2 and subsequent anti-IL-2 Ab pre-transplantation suppressed the proliferation of graft-infiltrating CD8+ T cells and donor-specific CTL activity, suggesting that many of allospecific CD8+ T cells in recipients are depleted due to IL-2-induced AICD as well as passive cell death caused by IL-2 withdrawal, the neutralizing anti-IL-2 Ab treatment. Indeed, donor MHC I DNA vaccination plus anti-IL-2 treatments without first administration of recombinant IL-2 did not significantly suppress donor-specific CTL activity (data not shown), indicating that AICD here is more important than the passive dell death for silencing donor-reactive CD8+ T cell response. The importance of deleting alloreactive CD8+ T cells for allograft survival was further confirmed by the finding that adoptive transfer of pre-sensitized syngeneic CD8+, but not CD4+, T cells abrogated the long-term allograft survival in our model. These results also support previous studies in which DST-mediated long-term transplant survival or tolerance was dependent on the peripheral deletion of donor-reactive CD8+ T cells [52], [53]. It remains to be defined why transfer of pre-sensitized CD4+ T cells is not sufficient to reverse the long-term allograft survival in this model.

## Materials and Methods

### Ethics Statement

All animal experiments were approved by the Animal Care and Use Committee of the University of Texas Health Science Center, Tyler, TX.

### Mice

Wild-type BALB/c (H-2^d^), C3H/HeJ (H-2^k^) and C57BL/6 (H-2^b^) mice were purchased from National Cancer Institute (NIH, Bethesda, MD, USA). IL-2-deficient mice were purchased from the Jackson Laboratory. All mice were housed in a specific pathogen-free environment.

### Plasmid Constructs and DNA Preparation

Murine major histocompatibility complex (MHC) class I (H-2K^d^) or class II (I-A^d^) cDNA was obtained by RT-PCR using total RNA isolated from the spleens of BALB/c mice, and subcloned into the eukaryotic expression vector pcDNA3.1+ (Invitrogen) at BamH I/Xho I sites, which carries the cytomegalovirus (CMV) and T7 promoter upstream of the cloned cDNA. The plasmid DNA vectors were prepared using a Qiagen Giga-prep kit (Qiagen, Valencia, CA) and are referred to as MHC I and MHC II respectively. The following PCR primers were used: H-2K^d^ forward primer was 5′-GGATCCATGGCACCCTGCACG-3′ and the reverse primer was 5′-CTCGAG TCACGCTAGAGAATGAGGG-3′; I-A^d^ forward primer was 5′-GGATCCATGCCGTGCAGTAGAGC-3′ and the reverse primer was 5′-CTCGAGTCATAAAGGCCCTGGG-3′.

### In Vivo Expression of Donor MHC Molecules and Tissue Immunofluorescence Staining

B6 mice received a single intramuscular (i.m.) injection (day 0) of 100 µg of plasmid DNA or empty vector in 100 µl of sterile saline. The injection sites were sampled at day 3 from each experimental group. The muscle samples were embedded in OTC and stored at −80°C until sectioning. Cryostat sections were fixed with acetone, washed, and blocked with 1∶20-diluted goat serum. Then slides were incubated with the primary Abs mouse anti-mouse H-2K^d^ and I-A^d^ (Biolegend) for 1 hour at 37°C, washed and incubated with 1∶200-diluted AlexaFluor 488- or 568-conjugated secondary Ab goat anti-mouse IgG (Invitrogen) at 4°C overnight. Slides were washed and air-dried. A coverslip was placed on the slide that was finally visualized under a fluorescence microscope.

### Immunization Protocols

B6 mice received (i.m.) 100 µg of plasmids encoding donor MHC I or MHC II gene or control vector in each quadricep muscle (day 0) alone or in combination with 1×10^4^ unit (1 µg) of recombinant mouse IL-2 (eBioscience) i.p. for four times (days 0, 2, 4 and 6), as described in **Figure 1**. Some mice that received MHC I vaccination plus recombinant IL-2 were treated with 50 µg of anti-mouse IL-2 mAb (Clone JES6-1A12, eBioscience) on days 8, 10 and 12, referred to as the group “MHC I + IL-2/anti-IL-2” (A). The immunization was boosted by repeating the same procedures once again in all groups two weeks after first immunization (day 14). In some groups, mice were treated with both “MHC I + IL-2/anti-IL-2” and subsequent “MHC II + IL-2”, once at a time, referred to as “A + B” (Figure 1). As controls, some mice also were treated with IL-2 alone or anti-IL-2 Ab alone at the same time points without immunization with the plasmid.

### Pancreatic Islet Transplantation

Islet donors were 8–10 week-old female Balb/C mice. Islet recipients were 8–10 week-old female C57BL/6 mice. Islets were isolated and transplanted into the subcapsular space of the right kidney of recipient mice (400 islets per recipient), as described previously [54]–[56]. Recipient mice were rendered diabetic by a single injection of streptozotocin (180 mg/kg) (Sigma) 10 days before transplantation. Primary graft function was defined as a blood glucose level under 200 mg/dl for 48 hours after transplantation. Islet graft rejection was defined as a rise in blood glucose to >300 mg/dl for three consecutive days after primary function.

### Histology

Kidney samples containing islet grafts were fixed in 10% formalin, embedded in paraffin, sectioned with a microtome, stained using haematoxylin/eosin (H&E), and observed for cell infiltration under a light microscope.

### Flow Cytometry

Spleen cells from immunized mice were stained with anti-CD4-PE, anti-CD25-FITC (BD Biosciences) and anti-FoxP3-APC Abs (eBioscience) and were analyzed by a FACSCalibur (BD Biosciences). In some experiments, graft-infiltrating cells were also stained for intracellular FoxP3 and BrdU (refer to BrdU labeling). To purify CD4+ and CD8+ T cells for adoptive transfer experiments, splenocytes from pre-sensitized B6 mice were stained with anti-CD4-PE, anti-CD8-FITC and anti-CD25-PerCP, and CD4+CD25− or CD8+CD25− cells were sorted out by FACSAria (BD Biosciences). The purity after sorting was typically >97%.

### Isolation of Tissue-Infiltrating Cells

Tissue-infiltrating cells were isolated as described in our previous publications [54], [57]. Briefly, the kidneys harboring islets were perfused *in situ* with heparinized 0.9% saline. They were then minced and digested at 37°C for 30 min in 20 ml RPMI-1640 medium containing 5% FCS and 350 u/ml collagenase (Sigma, St. Louis, MO). To clear the debris, cell suspensions were rapidly passed down 70 µm cell strainer, then mixed with Percoll solution (Sigma) to a concentration of 30%, and centrifuged at 2000 rpm for 15 minutes at room temperature. The pellet was re-suspended and analyzed for CTL activity or stained with Abs before FACS analysis.

### Analysis of T Cell Proliferation In Vivo by 5-Bromo-2′-Deoxyuridine (BrdU) Labeling

Recipient mice were pulsed i.p. with 0.8 mg of BrdU (Sigma) six days after transplantation. 24 hours later, renal graft-infiltrating cells were isolated and first stained with anti-CD4-PE or anti-CD8-PE. Cells were then fixed in 70% ethanol followed by 1% paraformaldehyde and incubated with 50 Units/ml of DNase I (Sigma). Cells were finally stained with anti-BrdU-FITC (BD Biosciences) and analyzed by a four-color FACSCalibur [54], [57].

### Cytotoxic T Lymphocyte (CTL) Activity

Graft-infiltrating cells were isolated and immediately assayed for *ex vivo* CTL activity against BALB/c targets, splenocytes. Allospecific CTL activity was measured by incubating the cells with either

Con A-activated (H-2^d^) BALB/c target spleen cells or third-party cells, LK35.2 (H-2^k^) (American Type Culture Collection) for 3 h. Target cells were labeled with calcein-AM (Molecular Probes), and calcein release was measured in a LS50B luminescence spectrometer (Perkin–Elmer) [58]. Experiments in which spontaneous calcein release was more than 25% of maximal release were excluded. Antigen-specific CTL activity was calculated according to the following formula: % specific lysis = 100 × [(sample release - spontaneous release)/(maximum release - spontaneous release)].

### Treatment of Mice with Depleting Anti-CD25 Ab and Rapamycin

To deplete CD4+CD25+ Treg cells, mice were treated with depleting anti-CD25 Ab (clone PC61, BioExpress, West Lebanon, NH)) at 0.25 mg every other day for four doses upon transplantation as described previously [54]. Over 80% of CD25+ T cells were depleted according FACS analysis. To provide mild immunosuppression, recipient mice were treated i.p. with low doses of rapamycin (0.2 mg/kg/day, Sigma) for seven consecutive days after islet transplantation.

### Statistical Analysis

The analysis of allograft survival data was performed using the Kaplan-Meier (Log-rank test). Comparison of means between groups was conducted using ANOVA. A value of p<0.05 was considered statistically significant.
